# Efficient plasmonic emission by the quantum Čerenkov effect from hot carriers in graphene

**DOI:** 10.1038/ncomms11880

**Published:** 2016-06-13

**Authors:** Ido Kaminer, Yaniv Tenenbaum Katan, Hrvoje Buljan, Yichen Shen, Ognjen Ilic, Josué J. López, Liang Jie Wong, John D. Joannopoulos, Marin Soljačić

**Affiliations:** 1 Department of Physics, Massachusetts Institute of Technology, 77 Massachusetts Avenue, Cambridge, Massachusetts 02139, USA; 2 Physics Department and Solid State Institute, Technion, Haifa 32000, Israel; 3 Department of Physics, University of Zagreb, Bijenička c. 32, 10000 Zagreb, Croatia; 4 Singapore Institute of Manufacturing Technology, 2 Fusionopolis Way, Innovis, Singapore 138634, Singapore

## Abstract

Graphene plasmons have been found to be an exciting plasmonic platform, thanks to their high field confinement and low phase velocity, motivating contemporary research to revisit established concepts in light–matter interaction. In a conceptual breakthrough over 80 years old, Čerenkov showed how charged particles emit shockwaves of light when moving faster than the phase velocity of light in a medium. To modern eyes, the Čerenkov effect offers a direct and ultrafast energy conversion scheme from charge particles to photons. The requirement for relativistic particles, however, makes Čerenkov emission inaccessible to most nanoscale electronic and photonic devices. Here we show that graphene plasmons provide the means to overcome this limitation through their low phase velocity and high field confinement. The interaction between the charge carriers flowing inside graphene and the plasmons enables a highly efficient two-dimensional Čerenkov emission, giving a versatile, tunable and ultrafast conversion mechanism from electrical signal to plasmonic excitation.

## Abstract (web summary)

Graphene plasmons have gained significant interest thanks to their high field confinement and low phase velocity. Here the authors show theoretically that charge carriers propagating in graphene can excite plasmons through a quantum Čerenkov emission process in two dimensions, in the form of plasmonic shock waves.

## Graphical abstract

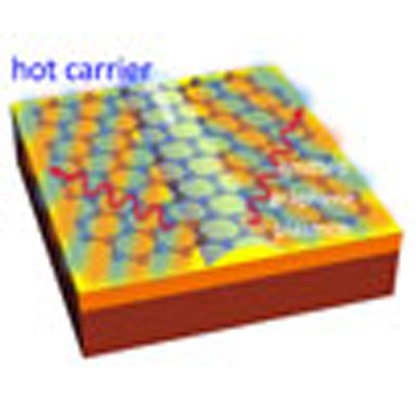

Achieving ultrafast conversion of electrical to optical signals at the nanoscale using plasmonics^1^,^2^ is a long-standing goal, owing to its potential to revolutionize electronics and allow ultrafast communication and signal processing. Given their strong field confinement, plasmonic systems combine the benefits of high frequencies (10^14^–10^15^ Hz) with those of small spatial scales, thus avoiding the limitation of conventional photonic systems. However, the realization of plasmonic sources that are electrically pumped, power efficient and compatible with current device fabrication processes (for example, complementary metal-oxide semiconductors (CMOS)), is a formidable challenge. In recent years, several groups have demonstrated the potential of surface plasmons as a platform for strong and ultrafast light–matter interaction^3^,^4^,^5^,^6^. Graphene's tunability and strong field confinement^7^,^8^,^9^,^10^ have motivated proposals for the use of graphene plasmons (GPs)^7^,^11^,^12^,^13^ in electrically pumped plasmonic sources^14^ and in the conversion of electrical energy into luminescence^15^,^16^,^17^.

Here we show that under proper conditions charge carriers propagating inside graphene can efficiently excite GPs, through a two-dimensional (2D) Čerenkov emission process. Graphene provides a platform in which the flow of charge alone is sufficient for Čerenkov radiation, eliminating the need for accelerated charge particles in vacuum chambers and opening up an opportunity for the study of the Čerenkov effect (ČE) and its applications, especially as a novel plasmonic source. Unlike other types of plasmon excitations, the 2D ČE manifests as a plasmonic shock wave, analogous to the conventional ČE that creates shockwaves in a three-dimensional (3D) medium. On a quantum mechanical level, this shockwave is reflected in the wavefunction of a single GP emitted from a single hot carrier.

## Results

### The mechanism of the graphene ČE

The mechanism of 2D ČE is enabled by two fundamental characteristics of graphene. On one hand, hot charge carriers moving with high velocities (up to the Fermi velocity *ν*_F_≈10^6^ m s^−1^) are considered possible, even in relatively large sheets of graphene (10 μm and more^18^). On the other hand, plasmons in graphene can have an exceptionally slow phase velocity, down to a few hundred times slower than the speed of light^7^,^9^,^19^. Consequently, velocity matching between charge carriers and plasmons is possible, enabling the emission of GPs from electrical excitations (hot carriers) at very high rates. This paves the way to devices using the ČE on the nanoscale, a prospect made even more attractive by the dynamic tunability of the Fermi level of graphene. For a wide range of parameters, the emission rate of GPs is significantly higher than the rates previously found for photons or phonons^7^,^20^,^21^, suggesting that taking advantage of the ČE enables near-perfect energy conversion from electrical energy to plasmons. Of course, it is impossible to reach perfect conversion due to other competing decay processes as discussed below. We further show that, contrary to expectations, plasmons can be created at energies above 2*E*_F_—thus exceeding energies attainable by photon emission—resulting in a plasmon spectrum that extends from terahertz to near-infrared frequencies and possibly into the visible range. Furthermore, we show that tuning the Fermi energy by external voltage can control the parameters (direction and frequency) of enhanced emission. This tunability also reveals regimes of backward GP emission and regimes of forward GP emission with low angular spread, emphasizing the uniqueness of ČE from hot carriers flowing in graphene. Surprisingly, we find that GP emission can also result from intraband transitions that are made possible by plasmonic losses. These kinds of transitions can become significant and might help explain several phenomena observed in graphene devices, such as current saturation^22^, high frequency radiation spectrum from graphene^17^,^23^ and the black body radiation spectrum that seems to relate to extraordinary high electron temperatures^24^.

**Figure 1:**
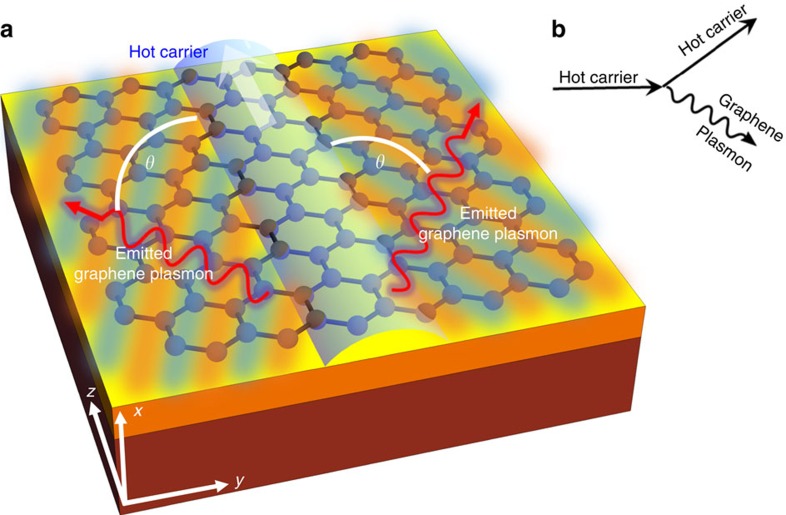
Illustration of the plasmon emission from charge carriers in graphene via a 2D Čerenkov process. (**a**) GP emission in graphene from a hot carrier flowing inside it. The hot carrier (white arrow marking a transparent-blue arch shape) excites GPs that propagate sideways (glowing red-blue bars) along the graphene surface (plotted on the yellow–orange–red substrate). The Čerenkov angle into which the GPs are emitted is denoted by *θ* (defined between the wiggling red arrows and the *z* axis, which is the direction of motion of the hot carrier). (**b**) A diagram describing the GP emission process from a hot carrier in graphene.

Recent studies^25^,^26^,^27^, which focus on cases of classical free charge particles moving outside graphene, have revealed strong Čerenkov-related GP emission resulting from the charge particle–plasmon coupling. In contrast, in this work we focus on the study of charge carriers inside graphene, as illustrated in Fig. 1a. For this purpose, we develop a quantum theory of ČE in graphene. As we shall see, our analysis of this system gives rise to a variety of novel Čerenkov-induced plasmonic phenomena.

The conventional threshold of the ČE in either 2D or 3D (*v*>*v*_p_) may seem unattainable for charge carriers in graphene, because they are limited by the Fermi velocity *v*≤*v*_*F*_, which is smaller than the GP phase velocity *v*_*F*_<*v*_p_, as shown by the random phase approximation calculations^19^,^28^. However, we show that quantum effects come into play, to enable these charge carriers to exceed the actual ČE threshold. Specifically, the actual ČE threshold for free electrons is shifted from its classically predicted value by the quantum recoil of electrons on photon emission^29^,^30^. Because of this shift, the actual ČE velocity threshold can in fact lie below the velocity of charge carriers in graphene, contrary to the conventional predictions. At the core of the modification of the quantum ČE is the linearity of the charge carrier energy–momentum relation (Dirac cone). Consequently, a careful choice of parameters (for example, Fermi energy or hot carrier energy) allows the ČE threshold to be attained—resulting in significant enhancements and high efficiencies of energy conversion from electrical to plasmonic excitation.

### The quantum theory of the ČE from hot carriers

The quantum ČE can be described as a spontaneous emission process of a charge carrier emitting into GPs, calculated by Fermi's golden rule^29^,^30^. In our case, the matrix elements must be obtained from the light–matter interaction term in the graphene Hamiltonian, illustrated by a diagram such as Fig. 1b. To model the GPs, we use the random phase approximation^19^,^28^,^31^,^32^, combined with a frequency-dependent phenomenological lifetime^19^ that takes into account additional loss mechanisms such as optical phonons and scattering from impurities in the sample (assuming graphene mobility of *μ*=2,000 cm^2^ V^−1^ s^−1^). This approach has been shown to give good agreement with experimental results^8^,^12^,^13^,^33^,^34^. The graphene sheet is in the *yz* plane and the charge carrier is moving in the *z* direction (Fig. 1a). For the case of low-loss GPs, the calculation reduces to the following integral (Lossy GPs are described later in this work—equation (6)).


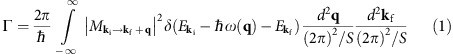



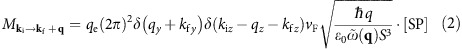


where 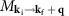 is the matrix element, *S* is the surface area used for normalization, *q*_e_ is the electric charge, *ɛ*_0_ is the vacuum permittivity, [SP] is the spinor–polarization coupling term and 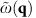 is the GP dispersion-based energy normalization term^35^ (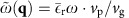, using the group velocity *v*_g_=∂*ω*/∂*q*). Supplementary Notes 1 and 2 elaborate on these definitions and describe the normalization (respectively). The GP momentum **q**=(*q*_*y*_, *q*_*z*_) satisfies 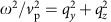, with the phase velocity *v*_p_=*v*_p_(*ω*) or *v*_p_(**q**) obtained from the plasmon dispersion relation as *v*_p_=*ω*/*q*. The momenta of the incoming (outgoing) charge carrier **k**_i_=(*k*_i*y*_, *k*_i*z*_) (**k**_f_=(*k*_f*y*_, *k*_f*z*_)) correspond to energies 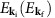 according to the conical momentum-energy relation 
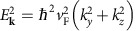
. The charge velocity is *v*=*E*_**k**_/|*ℏ***k**|, which equals a constant (*v*_F_). The only approximation in equation (2), henceforth, comes from the standard assumption of high GP confinement (free space wavelength/GP wavelength much larger than 1)^19^. Substituting equation (1) into equation (2) we obtain (denoting 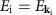):


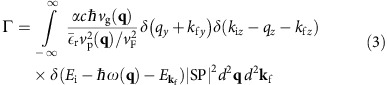


where 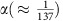 is the fine-structure constant, *c* is the speed of light and 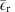 is the relative substrate permittivity obtained by averaging the permittivity on both sides of the graphene. We assume 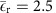 for all the figures. As material dispersion is neglected, all spectral features are uniquely attributed to the GP dispersion and its interaction with charge carriers, and not to any frequency dependence of the dielectrics. We further define the angle *ϕ* for the outgoing charge and *θ* for the GP, both relative to the *z* axis, which is the direction of the incoming charge. This notation allows us to simplify the [SP] for charge carriers inside graphene to |SP|^2^=cos^2^(*θ*−*ϕ*/2) or |SP|^2^=sin^2^(*θ*−*ϕ*/2) for intraband or interband transitions, respectively. The emission is restricted to two angles *θ*=±*θ*_Č_ , as reflected in the delta function in equation (3) (a clear signature of the ČE) and so we simplify the rate of emission to (see Supplementary Notes 3 and 4 for the complete derivation):


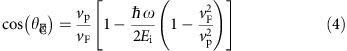



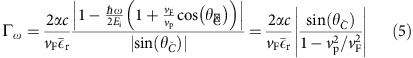


**Figure 2:**
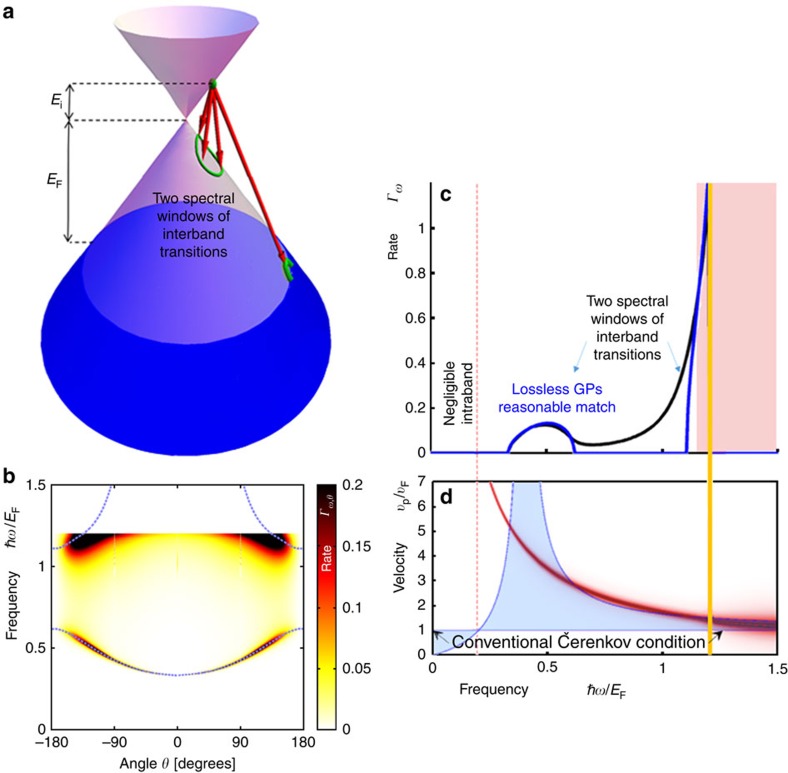
GP emission from hot carriers. (**a**) Illustration of the possible transitions. The hot carrier (green dot) has a range of potential transitions (red arrows) with distinct final states (green curves and circles), emitting plasmons that satisfy conservation of momentum and energy (corresponding to the height and angle of the red arrows). This way the cone geometry correlates the GP frequency and angle. The projection of these arrows to a 2D plane predicts the in-plane angle *θ* of the plasmonic emission, matching the (**b**) map of GP emission rate as a function of frequency and angle, equation (6). We find most of the GP emission around the dashed blue curves that are exactly found by the Čerenkov angle equation (4). (**c**) Spectrum of the ČE GP emission process, with the red regime marking the area of high losses (as in ref. 19). Black, emission spectrum with GP losses, equation (6). Blue, lossless emission approximation, equation (5). The thick orange line marks the spectral cutoff due to the Fermi sea, beyond which all states are occupied (therefore, *ℏω*<*E*_i_+*E*_F_). (**d**) Explaining the GP emission with the quantum ČE. The GP phase velocity is plotted as a red curve, with its thickness presenting the GP loss. The blue-shaded regime shows the range of allowed velocities according to the quantum ČE. We find enhanced GP emission in the frequencies for which the red curve crosses the blue regime, either directly or due to the curve thickness. The vertical dotted red line that crosses both **c** and **d** divides between interband to intraband transitions (exactly at *ℏω*=*E*_i_). At the parameters presented in this figure, there is only negligible intraband transitions (zero spectrum on the left of the dotted line). All figures are presented in normalized units, except for the angle shown in degrees. The hot carrier energy *E*_i_=0.2*E*_F_ and *n*_s_=3 × 10^13^ cm^−2^ (corresponding to *E*_F_=0.639 eV).

**Figure 3:**
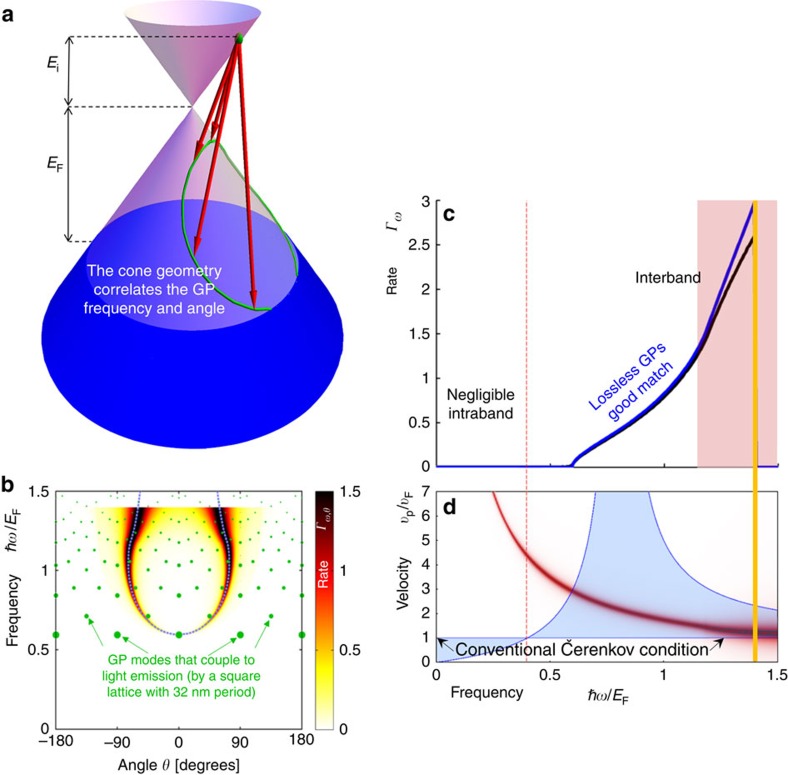
GP emission from hot carriers. Caption and notations same as in Fig. 2. The green dots in **b** show the GPs can be coupled out, as light, with each dot's size illustrating the strength of the coupling (in this case, the dots correspond to coupling out through a square lattice with a period of 32 nm in both dimensions). The hot carrier energy *E*_i_=0.4*E*_F_. *E*_F_ as in Fig. 2.

**Figure 4:**
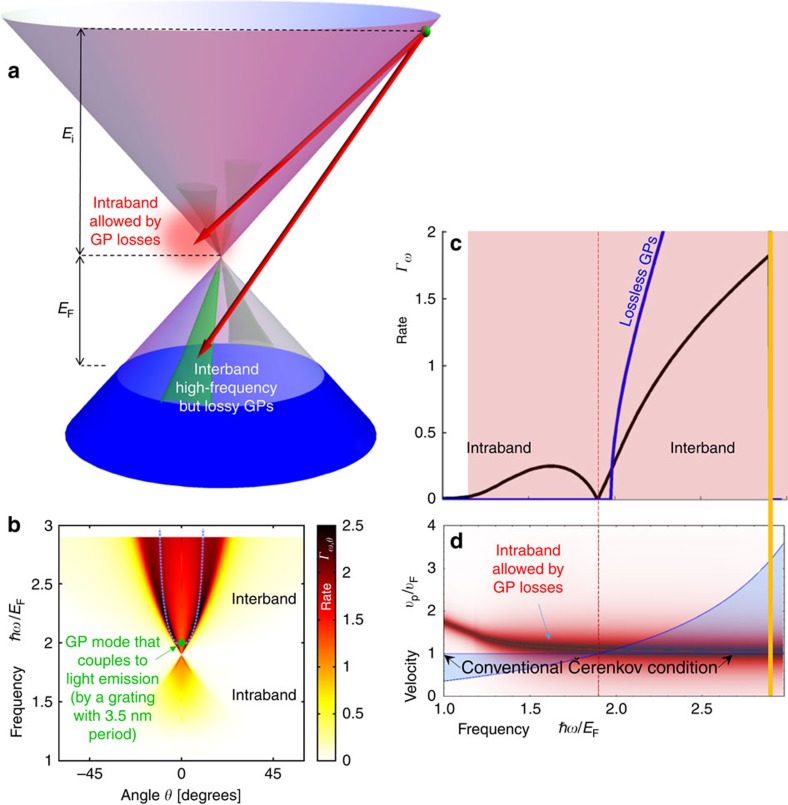
GP emission from hot carriers. Caption and notations same as in Fig. 2. Unlike conventional ČE, most of the emission occurs in the forward direction with a relatively low angular spread as is shown by **b**. The green dot shows that GPs a particular frequency can be coupled out as light (we assume a grating with period of 3.5 nm). For the parameters used here, ČE emission occurs due to intraband transitions that are becoming allowed by the GP losses, whereas high-frequency (such as *ℏω*>2*E*_F_) emission occurs due to interband transitions at areas of high GP losses. The hot carrier energy *E*_i_=1.9*E*_F_. *E*_F_ as in Fig. 2.

We note in passing that by setting *ℏ*→0 in the above expressions we recover the classical Čerenkov angle cos (*θ*_Č_)=*ν*_p_/*ν* and the classical 2D ČE, which can also be obtained from a purely classical electromagnetic calculation. However, although charge particles outside of graphene satisfy ℏ*ω*<<*E*_i_, making the classical approximation almost always exact^29^, the charges flowing inside graphene can have much lower energies, because they are massless. Consequently, the introduced ℏ terms in equations (4) and (5) modifies the conventional velocity threshold significantly, allowing ČE to occur for lower charge velocities. For example, although the conventional ČE requires charge velocity above the GP phase velocity (*v*>*v*_p_), equation (4) allows ČE below it and specifically requires the velocity of charge carriers in graphene (*v*=*v*_F_) to reside between 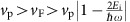. Physically, the latter case involves interband transitions made possible when graphene is properly doped: when the charge carriers are hot electrons (holes), interband ČE requires negatively (positively) doped graphene. Figures 2 and 3 demonstrate this interband ČE that indeed occurs for charge velocities below the conventional velocity threshold. More generally, the inequalities can be satisfied in two spectral windows simultaneously for the same charge carrier, owing to the frequency dependence of the GP phase velocity (shown by the intersection of the red curve with the blue regime in Fig. 2d). Moreover, part of the radiation (or even most of it, as in Fig. 2) can be emitted backward, which is considered impossible for ČE in conventional materials^36^,^37^. Several spectral cutoffs appear in Figs 2c, 3c and 4c, as seen by the range of non-vanishing blue spectrum. These can be found by substituting *θ*_Č_=0 in equation (4), leading to *ℏω*_cutoff_=2*E*_i_/(1±*v*_F_/*v*_p_), exactly matching the points where the red curves in Figs 2d, 3d and 4d cross the border of the blue regime. The upper most frequency cutoff marked by the thick orange line in Figs 2, 3, 4 occurs at *ℏω*=*E*_i_+*E*_F_ due to the interband transition being limited by the Fermi sea of excited states. Since *E*_i_ can be larger than *E*_F_, this implies that GP emission from electrical excitation can be more energetic than photon emission from a similar process (that is limited already by *ℏω*≲2*E*_F_). Finite temperature will broaden all cutoffs by the expected Fermi–Dirac distribution. However, for most frequencies, the GP losses are a more significant source of broadening.

### Including plasmonic losses into the graphene ČE

To incorporate the GP losses (as we do in all the figures), we modify the matrix elements calculation by including the imaginary part of the GP wavevector *q*_I_=*q*_I_(*ω*), derived independently for each point of the GP dispersion curve^19^. This is equivalent to replacing the delta functions in equation (3) by Lorentzians with 1/*γ* width, defining *γ*(*ω*)=*q*_R_(*ω*)/*q*_I_(*ω*). The calculation can be done partly analytically (Supplementary Note 3) yielding:


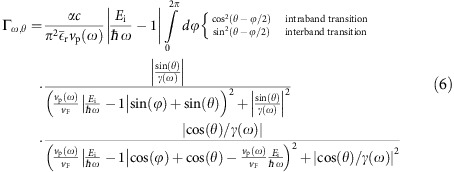


The immediate effect of the GP losses is the broadening of the spectral features as shown in Figs 2c, 3c and 4c. Still, the complete analytic theory of equations (4) and (5) matches very well with the exact graphene ČE. For example, regimes of enhanced emission in Figs 2b and 3b match with the blue dashed curves marked according to the Čerenkov angle formula of equation (4) (see Supplementary Note 4 for its derivation in the lossless limit). The presence of GP loss also opens up a regime of quasi-ČE that takes place when the charge velocity is very close to the Čerenkov threshold but does not exceed it. The addition of Lorentzian broadening then closes the gap, creating significant non-zero matrix elements that can lead to intraband GP emission (Fig. 4). This GP emission occurs even for hot electrons (holes) in positively (negatively) doped graphene, with the only change in Fig. 4 being that the upper frequency cutoff is instead shifted to *ℏω*≤*E*_i_−*E*_F_ (eliminating all interband transitions). The dip in the spectrum at the boundary between interband and intraband transitions (Fig. 4c) follows from the charge carriers' density of states being zero at the tip of the Dirac cone.

The interband ČE in Fig. 4 shows the possibility of emission of relatively high-frequency GPs, even reaching near-infrared and visible frequencies. These are interband transitions as in Figs 2 and 3, thus limited by *ℏω*≤*E*_i_+*E*_F_. Such a limit can get to a few eVs, because *E*_i_ is controlled externally by the mechanism creating the hot carriers (for example, *p*–*n* junction, tunnelling current in a heterostructure, scanning tunneling microscope (STM) tip, ballistic transport in graphene with high drain–source voltage and photoexcitation). GPs at near-infrared frequencies have already been shown to exist through direct and indirect experimental evidence^38^,^39^,^40^,^41^. The only fundamental limitation is the energy at which the graphene dispersion ceases to be conical (∼1 eV from the Dirac point^42^). Even then, our equations are only modified by changing the dispersion relations of the charge carrier and the GP, and therefore the graphene ČE should appear for *E*_*i*_ as high as (approximately) 3 eV^43^. The equations we presented are still valid, as they are written for a general dispersion relation, with *v*_*p*_(*ω*) and *γ*(*ω*) as parameters; thus, the basic predictions of the equations and the ČE features we describe will continue to hold regardless of the precise plasmon dispersion. For example, a recent paper^44^ suggests an alternative way of calculating GP dispersion, giving smaller GP phase velocities at high frequencies—this will lead to more efficient GP emission and another intraband regime that can occur without being mediated by the GP loss.

### Opportunities for experimental observation of the ČE in graphene

There exist several possible avenues for observing the quantum ČE in GPs, having to do with schemes for exciting hot carriers. For example, apart from photoexcitation, hot carriers have been excited from tunnelling current in a heterostructure^45^ and by a biased STM tip^38^. These methods can be used to generate hot carriers with narrow energy distributions but broad angular spreads. To show that many of the ČE features discussed in this study can be observed experimentally even with a broad angular distribution of hot carriers, Figs 2c and 3c plot the spectral distribution of the ČE averaged over the angular degree of freedom. Notably, the spectra contain clear signatures of the ČE (for example, the two distinct spectral peaks in Fig. 2c). This confirms that even with non-directional hot carriers plasmons with the distinctive attributes of Čerenkov radiation can be created and observed with established experimental means.

To generate hot carriers that are also directional, a possible method would be to apply a strong drain–source voltage across a graphene *p*–*n* junction^46^ or in other graphene devices with ballistic transport. This could be achieved by using concepts of graphene electron optics^47^, where recent experimental demonstrations have shown controlled and directional ballistic motion of hot carriers by specially designed gates and/or an external magnetic field^47^,^48^,^49^. The generation of directional hot carriers would facilitate the measurement of even more features of the ČE, such as the GP Čerenkov angle (as shown in Figs 2b, 3b and 4b). Yet, another intriguing approach for observing additional features of the graphene ČE could be exciting the hot carriers and measuring the generated ČE with the photon-induced near-field electron microscopy (PINEM)^50^,^51^, which might allow the visualization of the temporal dynamics of the Čerenkov emission. This approach is especially exciting, as the temporal dynamics of the ČE is expected to appear in the form of a plasmonic shockwave (as the conventional ČE appears as a shockwave of light).

**Figure 5:**
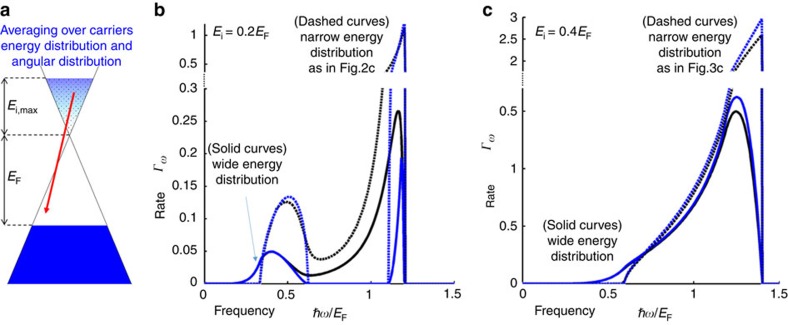
The spectrum of the Graphene ČE: hot carrier excitation energy having a wide versus narrow distribution. (**a**) Illustration of the distribution of hot carriers, which is taken to be an exponential multiplied by the linear electron density of states in graphene. The exponential decay is (**b**) 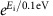 with maximum hot carrier energy of *E*_i,max_=0.2 eV corresponding to Fig. 2, or (**c**) 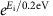 with maximum hot carrier energy of *E*_i,max_=0.4 eV corresponding to Fig. 3. In both **b** and **c** we plot the exact spectrum (integrating equation (6) over the energy distribution) in solid black and the lossless approximation (integrating equation (5) over the energy distribution) in solid blue. The dashed curves are for the respective cases of narrow energy distribution (matching Figs 2c and 3c). The Fermi energy *E*_F_ is as in Figs 2, 3, 4.

If the hot carriers have a broad distribution in both energy and direction, the spectral features will be partially smoothened but still present a clear signature of the graphene ČE. Hot carriers with a wide energy distribution (instead of a single *E*_i_), for instance, may be generated from a tunnelling current or *p*–*n* junction. The ČE spectrum corresponding to an arbitrary hot carrier excitation energy distribution is readily computed by integrating over a weighted distribution of ČE spectra for monoenergetic hot carriers. An example is presented in Fig. 5, where we assume the hot carriers have an exponential energy distribution (multiplied by the appropriate electron energy of states). This scenario can be readily created in a heterostructure^45^ having a sheet of graphene isolated from another conductor (possibly graphene as well), or by biasing an STM^38^ brought near the graphene surface. In both cases, the hot carriers tunnel through a potential barrier; thus, their distribution can be controlled by modifying the barrier thickness or the bias voltage. Figure 5a,b show that the spectral features are only partially smoothened and still present a clear signature of the graphene ČE. To give a rough estimate for energy conversion efficiency, it is noteworthy that the average GP energies in Fig. 5a,b are 0.93*E*_F_ and 1.1*E*_F_, respectively. Dividing by max(*E*_i_)+*E*_F_ (upper limit on the energy invested into a hot carrier), we get 77% and 78% (respectively) energy conversion efficiency. Here we assume that not more than one plasmon is emitted from each hot carrier—the efficiency is higher when GP emission from intraband trasitions is significant, as then multiple emissions from a single hot carrier are probable (as in Fig. 4).

Future studies could directly generalize our approach to regimes of higher rates of excitation of hot carriers, by solving for the steady-state occupation probability. Here we assume the hot carriers are sparse enough so that they relax to the Fermi sea after their GP emission without creating a significant occupation that would exclude the transitions of other hot carriers. Altogether, higher excitation rates are therefore expected to cause gradual saturation of the GP emission and thus reduce the conversion efficiency. Importantly, the conversion efficiency remains high even when the carriers' energy distribution is broad, as implied by the high ČE rate of emission for the representative values of *E*_*i*_ studied here (Figs 2, 3, 4 and 5 all show rates on the order of *Γ*_*ω*_∼1). Emission rates on the order of *Γ*_*ω*_∼1 are two to three orders of magnitude higher than those found in the conventional ČE in a 3D homogeneous dielectric, 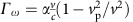. For example, in a bulk silicon nitride having 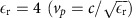, we find values ranging from *Γ*_*ω*_=0.0013 for *v*=0.6*c* to *Γ*_*ω*_=0.0055 for *v*→*c*. In graphene, the high ČE emission rate over a broad range of *E*_i_ owes itself to the low phase velocity and high confinement of GPs over a wide frequency range, which are properties unique to 2D plasmons.

Notably, the ČE emission of GPs can be coupled out as free-space photons by creating a grating or nanoribbons—fabricated in the graphene, in the substrate, or in a layer above it (see, for example, refs 39, 52, 53, 54)—with two arbitrarily chosen examples marked by the green dots in Figs 3b and 4b. Careful design of the coupling mechanism can restrict the emission to pre-defined frequencies and angles, with further optimization needed for efficient coupling. This clearly indicates that the GP emission, although usually considered as merely a virtual process, can be, in fact, completely real in some regimes, with the very tangible consequences of light emission in terahertz, infrared or possibly visible frequencies. Such novel sources of light could have promising applications due to graphene's dynamic tunability and small footprint (due to the small scale of GPs). Moreover, near-perfect conversion efficiency of electrical energy into photonic energy might be achievable due to the ČE emission rate dominating all other scattering processes. Lastly, unlike plasmonic materials such as silver and gold, graphene is especially exciting in this context, as it is CMOS compatible. Still, further research is needed in the design of gratings and/or cavities to minimize losses in the GP-to-photon conversion.

## Discussion

The high efficiency of GP emission through electrical excitation makes it very promising as a plasmonic source. The efficiency of coupling to the plasmons from free space, in contrast, is very low due to momentum mismatch (the GP momentum is two orders of magnitude higher than the momentum of a photon of the same frequency in free space). For regular plasmons, this mismatch is typically overcome using a grating (nanoribbons are also a viable option in graphene); however, this results in relatively low coupling efficiency. In general, the efficiency of out-coupling is similar to the efficiency of in-coupling, for example, 2% has been shown in the best cases of ref. 39. Yet, another approach to solve the momentum mismatch challenge is by using the nonlinear optical response of graphene^55^, for which coupling efficiencies approaching 10^−5^ have been reported. The prospect of attaining energy conversion efficiencies on the order of 77–78%, as in the example above, thus makes the quantum ČE from electrical excitation of hot carriers an exciting candidate for GP generation.

Furthermore, electrical excitation is interesting in itself due to its direct generation of radiation, without the need to use another radiation source (as in coupling lasers from free space). The unavoidable disadvantage of this scheme lies in the broad spectral range of the generated plasmons, compared with the highly monochromatic spectrum possible when using an external laser.

The hot carrier lifetime due to GP emission is defined by the inverse of the total rate of GP emission (integrating equation (5) or equation (6)) and can therefore be exceptionally short (down to a few femtosecond). Such short lifetimes are in general agreement with previous research on the subject (for example, see refs 20, 21, 56) that have shown electron–electron scattering as the dominant cooling process of hot carriers, unless hot carriers of relatively high energies (*E*_i_≈2*E*_F_ and above) are involved. In this latter case, one expects single-particle excitations to prevail over the contribution of the plasmonic resonances^57^. This is also in agreement with the fact that plasmons with high energies and momenta (in the electron-hole continuum, pink areas in Figs 2, 3, 4) are very lossy^7^,^19^. Additional factors that keep the ČE from attaining near-perfect conversion efficiency include other scattering processes such as acoustic and optical phonon scattering. Owing to the relatively long lifetime from acoustic phonon scattering (hundreds of femtoseconds to several picoseconds^7^), any deterioration due to this effect is not likely to be significant. Scattering by optical phonons is more significant for hot carriers above 0.2 eV, but its contribution is still about an order of magnitude smaller in our regime of interest^20^. Further research is necessary to optimize the emission process, including considerations of multi-plasmon emission and other higher-order effects.

Interestingly, the high rates of GP emission also conform to research of the reverse process—of plasmons enhancing and controlling the emission of hot carriers—that is also found to be particularly strong in graphene^58^,^59^. This might reveal unexplored relations between ČE and other novel ideas of graphene-based radiation sources that are based on different physical principles^60^,^61^,^62^,^63^,^64^.

It is also worth noting that Čerenkov-like plasmon excitations from hot carriers can be found in other condensed matter systems such as a 2D electron gas at the interface of semiconductors. Long before the discovery of graphene, such systems have demonstrated very high Fermi velocities (even higher than graphene's), while also supporting meV plasmons that can have slow phase velocities, partly due to the higher refractive indices possible in such low frequencies^65^. The ČE coupling, therefore, should not be unique to graphene. In many cases^66^,^67^, the coupling of hot carriers to bulk plasmons is even considered as part of the self-energy of the carriers, although the plasmons are then considered as virtual particles in the process. Nonetheless, graphene offers a unique opportunity where the Čerenkov velocity matching can occur at relatively high frequencies, with plasmons that have relatively low losses. Crucially, these differences are what makes the efficiency of the graphene ČE so high. Continued research into other 2D materials (for example, 2D silver and Beryllium^7^,^68^,^69^,^70^) may lead to materials with higher frequency, lower loss and higher confinement (lower phase velocity) than GPs. The prospect of higher frequency plasmons is especially exciting, as the ČE radiation intensity increases with frequency (explaining the bluish colour of conventional ČE).

We conclude with some very intriguing yet at this stage admittedly very speculative comments. Effects associated with the highly efficient emission of interband GPs and the unexpected emission of intraband GPs predicted by our quantum ČE theory may have already manifested themselves in current graphene experiments, even in ones that do not involve any optical measurement, such as transistor-based graphene devices^22^,^45^. For example, such GP emission could be a contributing factor to the effect of current saturation observed in graphene devices^22^, as large source–drain voltages can take graphene out of equilibrium and create hot carriers. When these hot carriers cross the energy threshold for significant GPs emission they lose energy abruptly, causing a sudden increase in resistivity. As another example, our graphene ČE might play a role in explaining the surprisingly high frequency of emitted light from graphene^17^,^23^,^24^, as GPs can couple out as light emission by surface roughness, impurities and so on. This hypothesis is encouraged by reports^17^ in which the measurements show characteristics typical of ČE variants, such as threshold values and power scaling behaviour that do not fit simple black body models. If our theory is indeed applicable here, then the extremely high-temperature estimates of the electron gas would need to be modified, to account for the contribution of GP emission in the high-frequency range of the observed spectrum. This would imply a lower black body radiation spectrum and thus lower graphene temperatures than otherwise expected^24^. Finally, as the GP energy can be higher than both *E*_i_ and *E*_F_, the ČE could form part of the explanation for the observed frequency up-conversion^23^, especially given that multi-plasmon effects are expected due to the high rate of the emission process. Of course, future detailed studies of the systems will be needed to verify the ČE connections proposed here.

### Data availability

The data supporting the findings of this study are available within the article and its Supplementary Information File.

## Additional information

**How to cite this article:** Kaminer, I. *et al.* Efficient plasmonic emission by the quantum Čerenkov effect from hot carriers in graphene. *Nat. Commun.* 7:11880 doi: 10.1038/ncomms11880 (2016).

## Supplementary Material

Supplementary Information Supplementary Figure 1, Supplementary Notes 1-4 and Supplementary References
