# Supplementary material for: Efficient plasmonic emission by the quantum Čerenkov effect from hot carriers in graphene
Source: Nat Commun. 2016 Jun 13;7:ncomms11880. doi: 10.1038/ncomms11880 (PMC4910017; doi:10.1038/ncomms11880)
Supplement: Supplementary Information — Supplementary Figure 1, Supplementary Notes 1-4 and Supplementary References [file ncomms11880-s1.pdf]

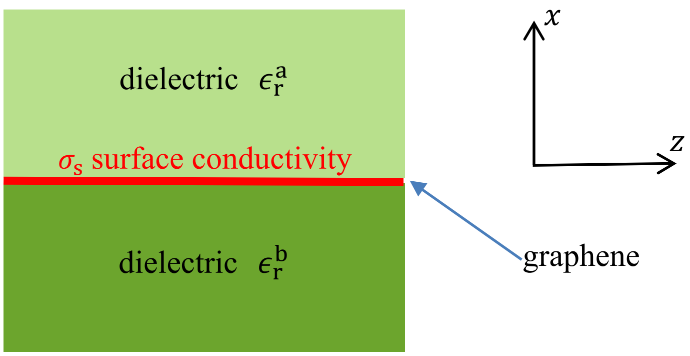

Supplementary Figure 1: Illustration of the graphene layer and its surrounding dielectrics.

Supplementary Note 1

Introducing graphene plasmons and their spinor-polarization coupling terms

We begin with the graphene Hamiltonian near the Dirac cone

$$H_{\text{graphene}} = \hbar v_F \boldsymbol{\sigma} \cdot \mathbf{k} \tag{1}$$

$$\boldsymbol{\sigma} = \left(\begin{pmatrix} 0 & 1 \\ 1 & 0 \end{pmatrix}, \begin{pmatrix} 0 & -i \\ i & 0 \end{pmatrix} \right) \tag{2}$$

using the same definitions as in the main text, with the Fermi velocity $v_F \approx 10^6\ m\ s^{-1}$ and the charge carrier momentum $\hbar\mathbf{k}$ satisfying a conical dispersion relation $E^2 = |v_F \hbar \mathbf{k}|^2$.

$\boldsymbol{\sigma}$ is a 2D vector of Pauli matrices. The eigenstates are [1]

$$\psi_{n,\mathbf{k}}(\mathbf{r}) = \frac{1}{\sqrt{S}} \begin{pmatrix} n \\ e^{i\varphi} \end{pmatrix} \frac{e^{i\mathbf{k}\cdot\mathbf{r}}}{\sqrt{2}} = \frac{1}{\sqrt{S}} \begin{pmatrix} n \\ e^{i\varphi} \end{pmatrix} \frac{e^{ik_z z + ik_y y}}{\sqrt{2}} \tag{3}$$

with the area S introduced for normalization purposes and the angle $\varphi = \measuredangle\mathbf{k} = \arctan(k_y/k_z)$.

The sign $n = 1$ ($n = -1$) denotes the conduction (valence) band.

To add an electromagnetic interaction (describes either a photon or a plasmon) we substitute

$$\hbar\mathbf{k} \to \hbar\mathbf{k} + q_e \mathbf{A} \tag{4}$$

where we choose the gauge for which the scalar potential is zero. This way we can write $\mathbf{A}$ directly from the in-plane electric field of the graphene plasmon $\mathbf{A} = -i\mathbf{E}/\omega$

This approach of treating an “effective photon” (in our case a plasmon) by the electron-photon QED interaction, derives from Ginzburg’s quantum description of the ČE [2], which has also been developed by many authors for different dispersion relations [3]

Next, we present the electric and magnetic fields that compose the graphene plasmon. This determines the polarization in the interaction Hamiltonian, and also gives the normalization of a single plasmon quantum state. Most interactions of graphene with an electromagnetic excitation consider free space photons, which has a negligible momentum in the plane of the graphene, so the interaction is typically fully described by a time harmonic term with no space dependence. In contrast, the graphene plasmon has large momentum that cannot be neglected. Moreover, the graphene plasmon has longitudinal polarization in the graphene plane, making the interaction term different from conventional light-matter interaction scenarios involving an electron and a photon.

The fields that compose the plasmon are obtained by matching boundary conditions of the electromagnetic fields that satisfy the Maxwell’s equations both above and below the graphene (denoted by a and b sides respectively, as illustrated in the Supplementary Fig.1):

$$\mathbf{E}^{\mathrm{a,b}} = \hat{x}E_{\perp}^{\mathrm{a,b}} + (\hat{z}\cos(\theta) + \hat{y}\sin(\theta))E_{\parallel}^{\mathrm{a,b}} \tag{5}$$

$$\mathbf{H}^{\mathrm{a,b}} = (\hat{y}\cos(\theta) - \hat{z}\sin(\theta))H_{\parallel}^{\mathrm{a,b}} \tag{6}$$

$$E_{\parallel}^{\mathrm{a,b}} = [FNC]e^{-i\omega t + iq_{\mathrm{y}}y + iq_{\mathrm{z}}z}e^{-k^{\mathrm{a,b}}x} \tag{7}$$

$$E_{\perp}^{\mathrm{a,b}} = E_{\parallel}^{\mathrm{a,b}}\frac{iq}{k^{\mathrm{a,b}}} \qquad H_{\parallel}^{\mathrm{a,b}} = i\omega E_{\parallel}^{\mathrm{a,b}}\frac{\epsilon_0\epsilon_{\mathrm{r}}^{\mathrm{a,b}}}{k^{\mathrm{a,b}}} \tag{8}$$

ϵ_0 is the vacuum permittivity, $\epsilon_{\mathrm{r}}^{\mathrm{a,b}}$ is the relative permittivity above (a) and below (b) the graphene sheet. $\perp$ denotes the polarization component that is perpendicular to the plane ($\hat{x}$) and $\parallel$ denotes the in-plane polarization component(s) (y, z plane). $k^{\mathrm{a,b}}$ is the decay rate perpendicular to the graphene sheet coming from the (imaginary part of the) wavevector in the $\hat{x}$ axis. The wavevector constituents are:

$$q_{\mathrm{y}} = q\sin(\theta) \qquad q_{\mathrm{z}} = q\cos(\theta) \tag{9}$$

$$\frac{q_{\mathrm{x}}^{\mathrm{a,b}}}{i} = k^{\mathrm{a,b}} = \pm\sqrt{q^2 - \left(\frac{\omega}{c}\right)^2\epsilon_{\mathrm{r}}^{\mathrm{a,b}}} \tag{10}$$

(for the geometry where a is above ($x > 0$) and b is below ($x < 0$) we have k^{a} real and positive while k^{b} real and negative). The field normalization coefficient [FNC] is an arbitrary complex coefficient according to the classical electromagnetic theory, but is given a fixed value below in the process of the field second quantization used for the quantum formalism of plasmon emission.

* In contrast with the above, the other polarization mode leads to a different plasmonic dispersion that does not have the same properties, and in particular does not have high confinement (and high momentum), therefore its interaction is negligible compared to the above polarization choice.

To determine the expression in the interaction Hamiltonian, we only need the in-plane polarization component of the electric field on the graphene plane ($x = 0$), giving:

$$\overline{\mathbf{A}}_{\parallel}^{x=0} = \widehat{\boldsymbol{q}} \frac{[\overline{\mathrm{FNC}}]}{-i\omega} e^{-i\mathbf{q}\cdot\mathbf{r}+i\omega t} \tag{11}$$

Therefore the interaction Hamiltonian has the spinor-polarization term $\boldsymbol{\sigma} \cdot \widehat{\boldsymbol{q}}$:

$$H_{\text{plasmon-interaction}} = q_e v_F \boldsymbol{\sigma} \cdot \overline{\mathbf{A}}_{\parallel}^{x=0} = q_e v_F \boldsymbol{\sigma} \cdot \widehat{\boldsymbol{q}} \frac{[\overline{\mathrm{FNC}}]}{-i\omega} e^{-i\mathbf{q}\cdot\mathbf{r}+i\omega t} \tag{12}$$

We define the spinor-polarization term [SP] by the following:

$$\psi_{n_{\mathrm{f}},\mathbf{k}_{\mathrm{f}}}^{\dagger} (\boldsymbol{\sigma} \cdot \hat{q}) \psi_{n_{\mathrm{i}},\mathbf{k}_{\mathrm{i}}} = [\mathrm{SP}] e^{i(\mathbf{k}_{\mathrm{i}}-\mathbf{k}_{\mathrm{f}})\cdot\mathbf{r}}/S \tag{13}$$

The indices i and f denote initial and final charge carrier in the process.

For a transition inside the same band $n_{\mathrm{f}} = n_{\mathrm{i}}$ (intraband) we get:

$$\begin{aligned} [\mathrm{SP}] &= \pm e^{i(\varphi_{\mathrm{f}}-\varphi_{\mathrm{i}})/2} \left[\frac{q_{\mathrm{z}}}{q} \cos\left(\tfrac{1}{2}(\varphi_{\mathrm{f}}+\varphi_{\mathrm{i}})\right) + \frac{q_{\mathrm{y}}}{q} \sin\left(\tfrac{1}{2}(\varphi_{\mathrm{f}}+\varphi_{\mathrm{i}})\right) \right] \\ &= \pm e^{i(\varphi_{\mathrm{f}}-\varphi_{\mathrm{i}})/2} \cos(\theta - (\varphi_{\mathrm{f}}+\varphi_{\mathrm{i}})/2) \end{aligned} \tag{14}$$

For a transition between the bands $n_{\mathrm{f}} = -n_{\mathrm{i}}$ (interband) we get:

$$\begin{aligned} [\mathrm{SP}] &= \pm i e^{i(\varphi_{\mathrm{f}}-\varphi_{\mathrm{i}})/2} \left[\frac{q_{\mathrm{y}}}{q} \cos\left(\tfrac{1}{2}(\varphi_{\mathrm{f}}+\varphi_{\mathrm{i}})\right) - \frac{q_{\mathrm{z}}}{q} \sin\left(\tfrac{1}{2}(\varphi_{\mathrm{f}}+\varphi_{\mathrm{i}})\right) \right] \\ &= \pm i e^{i(\varphi_{\mathrm{f}}-\varphi_{\mathrm{i}})/2} \sin(\theta - (\varphi_{\mathrm{f}}+\varphi_{\mathrm{i}})/2) \end{aligned} \tag{15}$$

Without loss of generality, we choose the incoming charge carrier along the z axis so that $\mathbf{k}_\mathrm{i} = \hat{z}E_\mathrm{i}/(\hbar v_\mathrm{F})$. The angles are then $\sphericalangle\mathbf{k}_\mathrm{i} = \varphi_\mathrm{i} = 0$ and $\sphericalangle\mathbf{k}_\mathrm{f} = \varphi_\mathrm{f} = \varphi$. This gives the expressions we use in the main text:

$$|\mathrm{SP}|^2 = \cos^2(\theta - \varphi/2) \qquad |\mathrm{SP}|^2 = \sin^2(\theta - \varphi/2) \tag{16}$$

Supplementary Note 2

The matrix elements and their normalization

The matrix elements describing the plasmon emission process (Fig.1 in the main text) is:

$$M_{\mathbf{k}_\mathrm{i}\to\mathbf{k}_\mathrm{f}+\mathbf{q}} = v_\mathrm{F} q_\mathrm{e} \iint\limits_S dydz\, \psi^\dagger_{n_\mathrm{f},\mathbf{k}_\mathrm{f}} \big(\boldsymbol{\sigma}\cdot\bar{\mathbf{A}}_\parallel^{x=0}\big)\psi_{n_\mathrm{i},\mathbf{k}_\mathrm{i}} \tag{17}$$

$$M_{\mathbf{k}_\mathrm{i}\to\mathbf{k}_\mathrm{f}+\mathbf{q}} = i\frac{q_\mathrm{e}v_\mathrm{F}}{\omega S}[\overline{\mathrm{FNC}}][\mathrm{SP}] \iint\limits_S dydz\, e^{i(\mathbf{k}_\mathrm{i}-\mathbf{k}_\mathrm{f}-\mathbf{q})\cdot\mathbf{r}} e^{i\omega t} \tag{18}$$

Next we find the normalization used in the quantization of the graphene plasmon field $[\overline{\mathrm{FNC}}]$, derived from the Poynting theorem for the energy carried by the field [3,4]. The energy stored in the electromagnetic field is:

$$U = \underbrace{\int dxdy}_{S} \int_{-\infty}^{\infty} dx |\mathbf{E}|^2 \frac{1}{2\omega}\frac{\partial}{\partial\omega}(\omega^2\varepsilon) \tag{19}$$

We assume no dispersion in the dielectrics on both sides of the graphene sheet. To calculate the energy we first have to consider the contribution of the graphene surface conductivity, because the energy stored in the 2D surface can be significant:

$$\varepsilon = \varepsilon_\mathrm{r}\varepsilon_0 + i\frac{\sigma}{\omega} = \varepsilon_\mathrm{r}\varepsilon_0 + i\frac{\sigma_\mathrm{s}}{\omega}\delta(x) = \varepsilon_\mathrm{r}\varepsilon_0 \underbrace{-2\frac{\epsilon_0\bar{\epsilon}_\mathrm{r}v_\mathrm{p}}{\omega}}_{\substack{lossless\ surface\\ for\ the\ energy\\ normalization}}\delta(x) \tag{20}$$

Substituting:

$$U = S\frac{1}{2\omega}\frac{\partial}{\partial\omega}(\omega^2\epsilon_0\epsilon_\mathrm{r}^\mathrm{a})\int_{\mathbf{0}}^{\infty} dx[|E_\parallel^\mathrm{a}|^2 + |E_\perp^\mathrm{a}|^2] + S\frac{1}{2\omega}\frac{\partial}{\partial\omega}(\omega^2\epsilon_0\epsilon_\mathrm{r}^\mathrm{b})\int_{-\infty}^{\mathbf{0}} dx\left[\left|E_\parallel^\mathrm{b}\right|^2 + \left|E_\perp^\mathrm{b}\right|^2\right]$$

$$-S\frac{1}{2\omega}\frac{\partial}{\partial\omega}\left(2\omega^2\frac{\epsilon_0\bar{\epsilon}_\mathrm{r}v_\mathrm{p}}{\omega}\right)\left[\left|E_\parallel^0\right|^2+\underbrace{|E_\perp^0|^2}_{=0}\right] \tag{21}$$

Where the parallel field component $E_\parallel^0 = E_\parallel^\mathrm{a}(x=0^+) = E_\parallel^\mathrm{b}(x=0^-)$, per the continuity relation, while the perpendicular field component is not continuous and averages to zero on the surface. Next, we apply an approximation that is typically used with graphene plasmons (the same approximation used in the main text): $k^\mathrm{a} \approx k^\mathrm{b} \approx q$. This is a good approximation because of the large confinement factor, or in other words, $q \gg \omega/c$ (free space wavelength >> graphene plasmon wavelength). It allows us to write:

$$U = 2\epsilon_0|\mathrm{FNC}|^2 S\frac{1}{2\omega}\frac{\partial}{\partial\omega}(2\omega^2\bar{\epsilon}_\mathrm{r})\int_{\mathbf{0}}^{\infty} dx e^{-2qx} - |\mathrm{FNC}|^2 S\frac{1}{2\omega}\frac{\partial}{\partial\omega}\left(\omega^2\frac{2\epsilon_0\bar{\epsilon}_\mathrm{r}v_\mathrm{p}}{\omega}\right) \tag{22}$$

$$U = 2\epsilon_0\bar{\epsilon}_\mathrm{r}|\mathrm{FNC}|^2 S 2\frac{1}{2q} - \epsilon_0\bar{\epsilon}_\mathrm{r}|\mathrm{FNC}|^2 S\frac{1}{\omega}\frac{\partial}{\partial\omega}(v_\mathrm{p}\omega) \tag{23}$$

$$U = \epsilon_0\bar{\epsilon}_\mathrm{r}|\mathrm{FNC}|^2 S\left[\frac{2}{q} - \frac{1}{\omega}\frac{\partial}{\partial\omega}(v_\mathrm{p}\omega)\right] = \epsilon_0\bar{\epsilon}_\mathrm{r}|FNC|^2 S\frac{v_\mathrm{p}}{q}\frac{\partial q}{\partial\omega} \tag{24}$$

The last step uses the following:

$$\frac{1}{\omega}\frac{\partial}{\partial\omega}(v_\mathrm{p}\omega) = \frac{1}{\omega}\frac{\partial}{\partial\omega}\left(\frac{\omega^2}{q}\right) = \frac{2}{q} - \frac{\omega}{q^2}\frac{\partial q}{\partial\omega} = \frac{2}{q} - \frac{v_\mathrm{p}}{q}\frac{\partial q}{\partial\omega} \tag{25}$$

At last, comparing the energy to $\hbar\omega$, we get the normalization coefficient $|\mathrm{FNC}|^2$:

$$\epsilon_0\bar{\epsilon}_\mathrm{r}|\mathrm{FNC}|^2 S\frac{v_\mathrm{p}}{q}\frac{\partial q}{\partial\omega} = \hbar\omega \tag{26}$$

With the definition $\widetilde{\omega} = \bar{\epsilon}_\mathrm{r}\omega\cdot\frac{v_\mathrm{p}}{v_\mathrm{g}} = \bar{\epsilon}_\mathrm{r}\omega\cdot v_\mathrm{p}\frac{\partial q}{\partial\omega}$ (as we had in the main text), we can write:

$$|\mathrm{FNC}|^2 = \omega^2\frac{\hbar q}{\widetilde{\omega}\epsilon_0 S} \tag{27}$$

Substituting [FNC] back to the matrix element we get:

$$M_{\mathbf{k}_\mathrm{i}\to\mathbf{k}_\mathrm{f}+\mathbf{q}} = i\frac{q_\mathrm{e}v_\mathrm{F}}{S}\sqrt{\frac{\hbar q}{\widetilde{\omega}\epsilon_0 S}}[\mathrm{SP}]\iint_S dydz\, e^{i(\mathbf{k}_\mathrm{i}-\mathbf{k}_\mathrm{f}-\mathbf{q})\cdot\mathbf{r}}e^{i\omega t} \tag{28}$$

Supplementary Note 3

Solving for the graphene Čerenkov effect with plasmonic losses

In this section we consider complex plasmonic momenta (taking losses into account) in the matrix elements, which we substitute into a Fermi golden rule calculation to find the rate of plasmon emission of the graphene ČE.

The y, z integrals in the matrix element give sinc functions that will become delta functions or Lorentzians when we take the normalization area S to infinity. To find the exact coefficient, we write S as $L_{\mathrm{y}}L_{\mathrm{z}}$ so:

$$\iint_S dydz\, e^{i(\mathbf{k}_{\mathrm{i}}-\mathbf{k}_{\mathrm{f}}-\mathbf{q})\cdot\mathbf{r}} = \int_{-L_{\mathrm{y}}/2}^{L_{\mathrm{y}}/2} dy \int_{-L_{\mathrm{z}}/2}^{L_{\mathrm{z}}/2} dz\, e^{i(\mathbf{k}_{\mathrm{i}}-\mathbf{k}_{\mathrm{f}}-\mathbf{q})\cdot\mathbf{r}}$$

$$= L_{\mathrm{y}}L_{\mathrm{z}}\mathrm{sinc}\left(\frac{L_{\mathrm{y}}}{2}\left(q_{\mathrm{y}} + k_{\mathrm{fy}}\right)\right)\mathrm{sinc}\left(\frac{L_{\mathrm{z}}}{2}\left(k_{\mathrm{iz}} - q_{\mathrm{z}} - k_{\mathrm{fz}}\right)\right) \tag{29}$$

The matrix element is modulo-squared hence we use the following delta function limit:

$$L_{\mathrm{z}}\left|\mathrm{sinc}\left(\frac{L_{\mathrm{z}}}{2}(k_{\mathrm{iz}} - q_{\mathrm{z}} - k_{\mathrm{fz}})\right)\right|^2 \xrightarrow[L_{\mathrm{z}}\to\infty]{} 2\pi\delta(k_{\mathrm{iz}} - q_{\mathrm{z}} - k_{\mathrm{fz}}) \tag{30}$$

We consider losses as an imaginary part added to the plasmon wavevector [5], denoting $q_{\mathrm{z}} = q_{\mathrm{Rz}} + iq_{\mathrm{Iz}}$ and $q_{\mathrm{y}} = q_{\mathrm{Ry}} + iq_{\mathrm{Iy}}$. In this case the limit of infinite area can be shown to give a Lorentzian.

$$\delta(k_{\mathrm{iz}} - q_{\mathrm{z}} - k_{\mathrm{fz}}) \to \frac{|q_{\mathrm{Iz}}|/\pi}{(k_{\mathrm{iz}} - q_{\mathrm{Rz}} - k_{\mathrm{fz}})^2 + |q_{\mathrm{Iz}}|^2} \tag{31}$$

Recalling the Fermi's golden rule (Eq.1 from the main text)

$$\Gamma = \underbrace{2}_{\substack{summing \\ over\ spin}}\frac{2\pi}{\hbar}\cdot\int_{-\infty}^{\infty}\left|M_{\mathbf{k}_{\mathrm{i}}\to\mathbf{k}_{\mathrm{f}}+\mathbf{q}}\right|^2\delta\big(E_{\mathrm{i}} - \hbar\omega(\mathbf{q}) - E_{\mathrm{f}}(\mathbf{k}_{\mathrm{f}})\big)\frac{d^2\mathbf{q}}{(2\pi)^2/S}\,\frac{d^2\mathbf{k}_{\mathrm{f}}}{(2\pi)^2/S} \tag{32}$$

We substitute the Lorentzians and get the following

$$\Gamma = \int_{-\infty}^{\infty}\frac{\alpha c\hbar\, v_{\mathrm{g}}(\mathbf{q})}{\bar{\epsilon}_{\mathrm{r}}\, v_{\mathrm{p}}^2(\mathbf{q})/v_{\mathrm{F}}^2}\delta\big(E_{\mathrm{i}} - \hbar\omega \pm E_{\mathbf{k}_{\mathrm{f}}}\big)|\mathrm{SP}|^2$$

$$\cdot\frac{\left|q_{\mathrm{Iy}}\right|/\pi}{\left(q_{\mathrm{Ry}} + k_{\mathrm{fy}}\right)^2 + \left|q_{\mathrm{Iy}}\right|^2}\frac{|q_{\mathrm{Iz}}|/\pi}{(k_{\mathrm{iz}} - q_{\mathrm{Rz}} - k_{\mathrm{fz}})^2 + |q_{\mathrm{Iz}}|^2}dq_{\mathrm{Rz}}dq_{\mathrm{Ry}}dk_{\mathrm{fz}}dk_{\mathrm{fy}} \tag{33}$$

v_{g} (v_{p}) is the group (phase) velocity of the graphene plasmom.

The integrals over the plasmon momentum are changed to

$$dq_{\mathrm{Rz}}dq_{\mathrm{Ry}} = \frac{q}{v_{\mathrm{g}}}d\theta d\omega = \frac{\omega/v_{\mathrm{p}}}{v_{\mathrm{g}}}d\theta d\omega \tag{34}$$

The integrals over the momentum of the outgoing charge carrier are used to solve the delta function over energy

$$\delta\left(E_{\mathrm{i}} - \hbar\omega \pm E_{\mathbf{k}_{\mathrm{f}}}\right)dk_{\mathrm{fz}}dk_{\mathrm{fy}} = \frac{|E_{\mathrm{i}} - \hbar\omega|}{(\hbar v_{\mathrm{f}})^2}d\varphi, \tag{35}$$

which restricts the energy of the outgoing charge carrier to $E_{\mathbf{k}_{\mathrm{f}}} = |E_{\mathrm{i}} - \hbar\omega|$. The absolute value is necessary because the charge carrier energies (incoming and outgoing) are defined relative to the tip of the Dirac cone. Therefore while intraband transitions will have $E_{\mathrm{i}} > \hbar\omega$, interband transitions can have $E_{\mathrm{i}} < \hbar\omega$. In both cases we can substitute:

$$k_{\mathrm{fz}} = \frac{|E_{\mathrm{i}}-\hbar\omega|}{\hbar v_{\mathrm{F}}}\cos(\varphi) \qquad k_{\mathrm{fy}} = \frac{|E_{\mathrm{i}}-\hbar\omega|}{\hbar v_{\mathrm{F}}}\sin(\varphi) \tag{36}$$

Eventually obtaining Eq.6 from the main text (defining $\gamma(\omega) = q_{\mathrm{R}}(\omega)/q_{\mathrm{I}}(\omega)$):

$$\begin{aligned} \Gamma_{\omega,\theta} = {} & \frac{\alpha c}{\pi^2 \bar{\epsilon}_{\mathrm{r}} v_{\mathrm{p}}(\omega)}\left|\frac{E_{\mathrm{i}}}{\hbar\omega} - 1\right| \int_0^{2\pi} d\varphi \quad \begin{cases} \cos^2(\theta - \varphi/2) & \mathit{intraband\ transition} \\ \sin^2(\theta - \varphi/2) & \mathit{interband\ transition} \end{cases} \\ & \cdot \frac{\left|\frac{\sin(\theta)}{\gamma(\omega)}\right|}{\left(\frac{v_{\mathrm{p}}(\omega)}{v_{\mathrm{F}}}\left|\frac{E_{\mathrm{i}}}{\hbar\omega} - 1\right|\sin(\varphi) + \sin(\theta)\right)^2 + \left|\frac{\sin(\theta)}{\gamma(\omega)}\right|^2} \\ & \cdot \frac{|\cos(\theta)/\gamma(\omega)|}{\left(\frac{v_{\mathrm{p}}(\omega)}{v_{\mathrm{F}}}\left|\frac{E_{\mathrm{i}}}{\hbar\omega} - 1\right|\cos(\varphi) + \cos(\theta) - \frac{v_{\mathrm{p}}(\omega)}{v_{\mathrm{F}}}\frac{E_{\mathrm{i}}}{\hbar\omega}\right)^2 + |\cos(\theta)/\gamma(\omega)|^2} \end{aligned} \tag{37}$$

Supplementary Note 4

The graphene Čerenkov effect in its lossless limit and compared to the conventional theory

We consider the limit case of low losses, since then further analytical simplifications can be achieved. By taking the delta function limit of the Lorentzians Eq.1 translates into

$$\Gamma = \int d^2\mathbf{q}\,\frac{\alpha c\hbar\, v_{\mathrm{g}}(\mathbf{q})}{\bar{\epsilon}_{\mathrm{r}}\, v_{\mathrm{p}}^2(\mathbf{q})/v_{\mathrm{F}}^2}\,\delta\left(E_{\mathrm{i}} - \hbar\omega(\mathbf{q}) \pm E_{\mathbf{k}_{\mathrm{f}}}\right)|\mathrm{SP}|^2 \tag{38}$$

$$\Gamma_\omega = \int_0^{2\pi} d\theta \frac{\alpha c\, \hbar\omega/v_{\mathrm{p}}(\omega)}{\bar{\epsilon}_{\mathrm{r}}\, v_{\mathrm{p}}^2(\omega)/v_{\mathrm{F}}^2} \delta\left(E_{\mathrm{i}} - \hbar\omega \pm E_{\mathbf{k}_{\mathrm{f}}}(\theta,\omega)\right) |\mathrm{SP}|^2 \tag{39}$$

Changing the integration variable θ to $E_{\mathbf{k}_{\mathrm{f}}}$ gives the following Jacobian

$$2\left|\frac{1-\frac{\hbar\omega}{E_{\mathrm{i}}}}{\hbar\omega\frac{v_{\mathrm{F}}}{v_{\mathrm{p}}}\sin(\theta_{\check{\mathrm{C}}})}\right|, \tag{40}$$

which we substitute back to get:

$$\Gamma_\omega = \frac{2\alpha c v_{\mathrm{F}}}{\bar{\epsilon}_{\mathrm{r}} v_{\mathrm{p}}^2(\omega)}\left|\frac{1-\frac{\hbar\omega}{E_{\mathrm{i}}}}{\sin(\theta_{\check{\mathrm{C}}})}\right| |\mathrm{SP}|^2 \tag{41}$$

The delta function forces the conservation of energy $E_{\mathbf{k}_{\mathrm{f}}}(\theta,\omega) = |E_{\mathrm{i}} - \hbar\omega|$, which gives the modified Čerenkov angle:

$$E_{\mathrm{i}} - \hbar\omega = \pm v_{\mathrm{F}}\hbar|\mathbf{k}_{\mathrm{f}}| \tag{42}$$

$$(E_{\mathrm{i}} - \hbar\omega)^2 = (\hbar v_{\mathrm{F}})^2[|\mathbf{k}_{\mathrm{i}}|^2 + |\mathbf{q}|^2 - 2\mathbf{k}_{\mathrm{i}}\cdot\mathbf{q}] \tag{43}$$

$$(E_{\mathrm{i}} - \hbar\omega)^2 = E_{\mathrm{i}}^2 + \left(\hbar\omega v_{\mathrm{F}}/v_{\mathrm{p}}\right)^2 - 2E_{\mathrm{i}}\hbar\omega v_{\mathrm{F}}/v_{\mathrm{p}}\cos(\theta_{\check{\mathrm{C}}}) \tag{44}$$

$$2E_{\mathrm{i}}\hbar\omega\frac{v_{\mathrm{F}}}{v_{\mathrm{p}}}\cos(\theta_{\check{\mathrm{C}}}) = (\hbar\omega)^2\left(\left(\frac{v_{\mathrm{F}}}{v_{\mathrm{p}}}\right)^2 - 1\right) + 2E_{\mathrm{i}}\hbar\omega \tag{45}$$

$$\frac{v_{\mathrm{F}}}{v_{\mathrm{p}}}\cos(\theta_{\check{\mathrm{C}}}) = 1 + \frac{\hbar\omega}{2E_{\mathrm{i}}}\left(\left(\frac{v_{\mathrm{F}}}{v_{\mathrm{p}}}\right)^2 - 1\right) \tag{46}$$

Therefore the equation for the Čerenkov angle in the quantum 2D ČE (Eq.4 from the main text):

$$\cos(\theta_{\check{\mathrm{C}}}) = \frac{v_{\mathrm{p}}}{v_{\mathrm{f}}}\left[1 - \frac{\hbar\omega}{2E_{\mathrm{i}}}\left(1 - \frac{v_{\mathrm{F}}^2}{v_{\mathrm{p}}^2}\right)\right] \tag{47}$$

Notice that when taking $\hbar \to 0$ (or $E_{\mathrm{i}} \gg \hbar\omega$), we get the well-known equation $\cos(\theta_{\check{\mathrm{C}}}) = v_{\mathrm{p}}/v_{\mathrm{F}}$ for the conventional Čerenkov angle [3].

To find the total rate of plasmon emission we need to explicitly find the spinor-polarization term $|\mathrm{SP}|^2$ by substituting the angles of the plasmon ($\theta_{\check{\mathrm{C}}}$) and of the outgoing charge carrier (φ). The latter can be found from the delta functions on the momenta in the y axis $\delta\left(q_{\mathrm{y}} + k_{\mathrm{fy}}\right)$:

$$\sin(\varphi) = -\frac{\sin(\theta)}{\left|\frac{E_{\mathrm{i}}}{\hbar\omega} - 1\right|}\frac{v_{\mathrm{F}}}{v_{\mathrm{p}}}, \tag{48}$$

or from the delta function on the momenta in the z axis $\delta(k_{\mathrm{iz}} - q_{\mathrm{z}} - k_{\mathrm{fz}})$:

$$\cos(\varphi) = \frac{1}{\left|\frac{\hbar\omega}{E_{\mathrm{i}}} - 1\right|} - \frac{\cos(\theta)}{\left|\frac{E_{\mathrm{i}}}{\hbar\omega} - 1\right|}\frac{v_{\mathrm{F}}}{v_{\mathrm{p}}} \tag{49}$$

We write $|\mathrm{SP}|^2$ in the following way capturing both interband and intraband transitions:

$$|\mathrm{SP}|^2 = \frac{1 \pm \cos(2\theta_{\check{\mathrm{C}}} - \varphi)}{2}, \tag{50}$$

which we can translate to the following form:

$$|\mathrm{SP}|^2 = \frac{v_{\mathrm{p}}^2}{v_{\mathrm{F}}^2}\frac{\left|1 - \frac{\hbar\omega}{2E_{\mathrm{i}}}\left(1 + \frac{v_{\mathrm{F}}}{v_{\mathrm{p}}}\cos(\theta_{\check{\mathrm{C}}})\right)\right|}{\left|1 - \frac{\hbar\omega}{E_{\mathrm{i}}}\right|} \tag{51}$$

or further simplify to get the following:

$$|\mathrm{SP}|^2 = \frac{v_{\mathrm{p}}^2}{v_{\mathrm{F}}^2}\frac{1}{\left|1 - \frac{\hbar\omega}{E_{\mathrm{i}}}\right|}\frac{\sin^2(\theta_{\check{\mathrm{C}}})}{1 - v_{\mathrm{p}}^2/v_{\mathrm{F}}^2} \tag{52}$$

Giving the equation for the rate of plasmon emission per unit frequency from a single hot carrier in graphene (Eq.5)

$$\Gamma_\omega = \frac{2\alpha c}{v_{\mathrm{F}}\bar{\epsilon}_{\mathrm{r}}}\frac{\left|1 - \frac{\hbar\omega}{2E_{\mathrm{i}}}\left(1 + \frac{v_{\mathrm{F}}}{v_{\mathrm{p}}}\cos(\theta_{\check{\mathrm{C}}})\right)\right|}{|\sin(\theta_{\check{\mathrm{C}}})|} = \frac{2\alpha c}{v_{\mathrm{F}}\bar{\epsilon}_{\mathrm{r}}}\left|\frac{\sin(\theta_{\check{\mathrm{C}}})}{1 - v_{\mathrm{p}}^2/v_{\mathrm{F}}^2}\right| \tag{53}$$

Notice that in the limit of $\hbar \to 0$ (or $E_{\mathrm{i}} \gg \hbar\omega$) we get the following

$$\Gamma_\omega = \frac{2\alpha c}{v_{\mathrm{F}}\bar{\epsilon}_{\mathrm{r}}}\frac{1}{|\sin(\theta_{\check{\mathrm{C}}})|} = \frac{2\alpha c}{v\bar{\epsilon}_{\mathrm{r}}}\frac{1}{\sqrt{1 - v_{\mathrm{p}}^2/v^2}}, \tag{54}$$

which is the classical limit of the Čerenkov Effect for a free charge particle (relativistic or not) moving outside graphene and parallel to it with velocity v (here there are no quantum corrections hence the regular Čerenkov threshold $v > v_{\mathrm{p}}$ is required for the emission of plasmons). The same expression can be derived from the Maxwell equations.

Supplementary references

[1] Neto, A. C., Guinea, F., Peres, N. M. R., Novoselov, K. S., & Geim, A. K. The electronic properties of graphene. *Reviews of Modern Physics*, **81**, 109 (2009).

[2] Ginzburg, V. L., Quantum Theory of Radiation of Electron Uniformly Moving in Medium, *Zh. Eksp. Teor. Fiz.* **10**, 589-600 (1940); *J. Phys. USSR* **2**, 441-452 (1940).

[3] Harris, E. G., *A pedestrian approach to quantum field theory* (John Willey and Sons, Inc., New York, London, 1972).

[4] Landau, L. D. and Lifshitz, E. M. *Electrodynamics of Continuous Media* (Addison-Wesley, Reading, Mass., 1960)

[5] Jablan, M., Buljan, H., and Soljacic, M., Plasmonics in graphene at infrared frequencies, *Phys. Rev. B* **80**, 245435 (2009).
